# Association of smoking and cardiometabolic parameters with albuminuria in people with type 2 diabetes mellitus: a systematic review and meta-analysis

**DOI:** 10.1007/s00592-019-01293-x

**Published:** 2019-02-24

**Authors:** Debasish Kar, Clare Gillies, Mintu Nath, Kamlesh Khunti, Melanie J. Davies, Samuel Seidu

**Affiliations:** 1 Diabetes Research Centre, Univerisity of Leicester, Leicester, UK; 2 Academic Unit of Diabetes and Endocrinology, University of Sheffield, Sheffield, UK

**Keywords:** Type 2 diabetes mellitus, Albuminuria, Smoking

## Abstract

**Aims:**

Smoking is a strong risk factor for albuminuria in people with type 2 diabetes mellitus (T2DM). However, it is unclear whether this sequela of smoking is brought about by its action on cardiometabolic parameters or the relationship is independent. The aim of this systematic review is to explore this relationship.

**Methods:**

Electronic databases on cross-sectional and prospective studies in Medline and Embase were searched from January 1946 to May 2018. Adult smokers with T2DM were included, and other types of diabetes were excluded.

**Results:**

A random effects meta-analysis of 20,056 participants from 13 studies found that the odds ratio (OR) of smokers developing albuminuria compared to non-smokers was 2.13 (95% CI 1.32, 3.45). Apart from smoking, the odds ratio of other risk factors associated with albuminuria were: age 1.24 (95% CI 0.84, 1.64), male sex 1.39 (95% CI 1.16, 1.67), duration of diabetes 1.78 (95% CI 1.32, 2.23), HbA1c 0.63 (95% CI 0.45, 0.81), SBP 6.03 (95% CI 4.10, 7.97), DBP 1.85 (95% CI 1.08, 2.62), total cholesterol 0.06 (95% CI − 0.05, 0.17) and HDL cholesterol − 0.01 (95% CI − 0.04, 0.02), triglyceride 0.22 (95% CI 0.12, 0.33) and BMI 0.40 (95% CI 0.00–0.80). When the smoking status was adjusted in a mixed effect meta-regression model, the duration of diabetes was the only statistically significant factor that influenced the prevalence of albuminuria. In smokers, each year’s increase in the duration of T2DM was associated with an increased risk of albuminuria of 0.19 units (95% CI 0.07, 0.31) on the log odds scale or increased the odds approximately by 23%, compared to non-smokers. Prediction from the meta-regression model also suggested that the odds ratios of albuminuria in smokers after a diabetes duration of 9 years and 16 years were 1.53 (95% CI 1.10, 2.13) and 5.94 (95% CI 2.53, 13.95), respectively.

**Conclusions:**

Continuing to smoke and the duration of diabetes are two strong predictors of albuminuria in smokers with T2DM. With a global surge in younger smokers developing T2DM, smoking cessation interventions at an early stage of disease trajectory should be promoted.

**Electronic supplementary material:**

The online version of this article (10.1007/s00592-019-01293-x) contains supplementary material, which is available to authorized users.

## Introduction

Smokers with T2DM are disproportionately affected by premature cardiovascular events. A recent systematic review of over 1 million people revealed that smokers with T2DM were approximately 50% more likely to die prematurely with cardiovascular events, compared to non-smokers [1]. However, the precise underlying cause for this heightened cardiovascular mortality remains unexplored. Smoking exacerbates insulin resistance, and adversely affects some cardiometabolic risk factors in T2DM including HbA1c, HDL cholesterol and arterial blood pressure [2]. Surprisingly, however, smoking cessation does not appear to confer any substantial cardiovascular risk reduction for up to 10 years in people with diabetes, compared to 3 years in people without [3]. Indeed, the World Health Organization (WHO) Multinational Study of Vascular Disease in Diabetes (MSVDD) demonstrated that the risk of cardiovascular mortality in people with diabetes remains up to 50% higher in recent quitters (1–9 years), compared to non-smokers [4]. This incongruous relationship between smoking cessation and mortality suggests that there may be some additional risk factor/s that contribute to a higher cardiovascular risk in recent quitters, which might not be reversed by short-term abstinence from smoking.

Albuminuria is an early indicator of both micro-, and macrovascular involvements in diabetes [5, 6] and the progression of albuminuria is a reliable marker for the extent of vascular perturbation [7]. Aggressive management of traditional risk factors such as glucose, blood pressure and lipid profile has not shown consistent benefit particularly when proteinuria is already established [8]. On the other hand, multifactorial interventions including smoking cessation at an early stage of disease trajectory have shown promising potential for the reversal of microalbuminuria and improved cardiovascular outcome [9]. However, conventional risk stratification score derived from the HbA1c, blood pressure and lipid profile may underestimate the influence of life style factors such as obesity and smoking on albuminuria during this crucial stage of disease trajectory. With a global surge of younger people developing metabolic syndrome and T2DM, it is pivotal to explore how best they can be protected from albuminuria which not only heralds incipient diabetic nephropathy but also poses a higher risk for premature cardiovascular complications. The aim of this systematic review and meta-analysis is to elucidate how smoking impacts upon the prevalence of albuminuria and how this relationship is influenced by cardiovascular risk factors such as age, male sex, duration of diabetes, HbA1c, blood pressure, lipid profile and body mass index (BMI).

## Materials and methods

### Search strategy and selection criteria

**Fig. 1:**
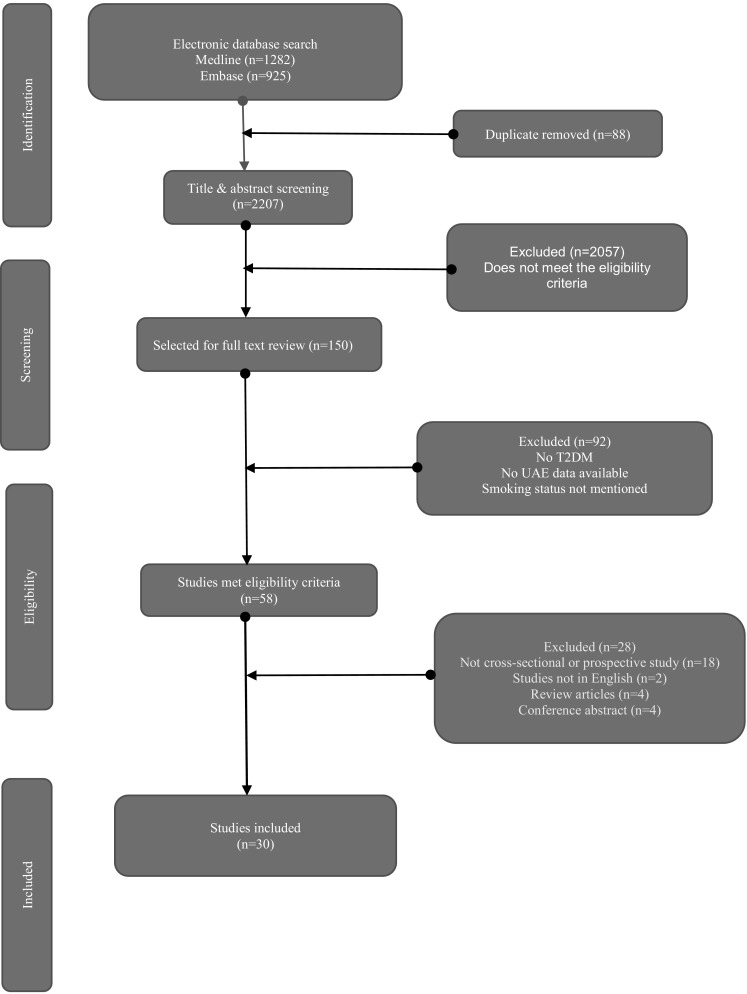
PRISMA flow chart

For this systematic review and meta-analysis, we conducted a comprehensive search on Medline and Embase electronic databases from their inceptions to May 2018. The keywords used for the searches were: “type 2 diabetes”, “smoking”, “microalbuminuria” or “macroalbuminuria” or “albuminuria” or “proteinuria” in the title, abstract and keywords; the result was then combined using the Boolean operator “AND”. Additionally, we searched the references of the included studies to identify further suitable studies for inclusion. We followed the Preferred Reporting Items for Systematic Reviews and Meta-analysis Protocol (PRISMA-P) 2015 guidelines [10] (Fig. 1). We published the protocol in the International Prospective Register for Systematic Reviews (PROSPERO) database (CRD 42018090637). The full search strategy is in Supplementary material 1.

The inclusion criteria were studies reporting urinary albumin excretion (UAE) in adults (> 18 years) with T2DM. T2DM was defined as a condition affecting people’s blood sugar level which was then diagnosed by healthcare professionals and treated with diet, lifestyle interventions, oral medication or injectable therapy. People with type 1 diabetes mellitus (T1DM), steroid-induced diabetes, diabetes insipidus, and late auto-immune diabetes of adults (LADA) were excluded, but maturity-onset diabetes of the young (MODY) were included. Smokers were defined as self-reported cigarette smokers for at least a year after being diagnosed with T2DM. For this study, albuminuria was defined as urinary albumin creatinine ratio (ACR) > 20 mg/gm or > 2.5 mg/mmol in male or > 3.5 mg/mmol in female (KDIGO—Kidney Disease Improving Global Outcome guidelines http://kdigo.org) [11]. Total cholesterol and HDL were converted to mmol/l if they were reported in mg/dl (mg/dl = ÷ 38.67 mmol/l), and TG (mg/dl = ÷ 88.57). HbA1c was expressed in both IFCC unit (mmol/mol) and DCCT unit (%). Blood pressure was expressed in mm of Hg and BMI was expressed as kg/m^2^.

Studies in English language or translated into the English language were accepted for inclusion. Observational prospective and cross-sectional studies were included for this review. Two investigators (DK and CLG) independently screened the articles using the inclusion and exclusion criteria. Any disagreement between the two investigators was resolved either by consensus or by consulting with a third investigator (SS). Included studies were selected by reviewing the titles and abstracts on electronic databases search. Additionally, hand searches were carried out from the references of the included studies.

### Data analysis

Data extraction was conducted using a predesigned data extraction template—study name, year of publication, country of study, study design, number of participants, mean age, smoking status of the participants, and presence or absence of albuminuria (Table 1).

**Table 1:** Characteristics of the included studies (baseline data for prospective studies unless stated otherwise)

Study included name/ID	Study design	Country	Mean age (years)	Sex (% male)	Number of participants (*n*)	Smoking status (*n*)			Albuminuria (*n*)		Mean duration of DM	Mean HbA1c	Mean SBP
						S	NS	Q	Yes	No	(years)	mmol/mol(%)	(mm of Hg)
Chuahirun et al., 2003 [50]/08	Prospective	USA	45	55	33	13	20	NS	NS	NS	NS	92 (10.6)	NS
Chuahirun et al., 2004 [51]/09	Prospective	USA	45	54	84	31	53	NS	46	38	5	95 (10.8)	115
Chuahirun et al., 2004 [60]/09	Prospective	USA	49	50	157	69	88	NS	112	45	5	54 (7.06)	132
Ikeda et al., 1996 [52]/15	Cross-sectional	Japan	62	100	142	81	40	21	58	84	NS	62 (7.8)	137
Tseng et al., 2010 [53]/28	Prospective	Taiwan	58	55	519	199	320	NS	240	279	10	64 (8.0)	132
Voulgari et al., 2011 [54]/29	Prospective	Greece	56	50	193	73	NS	120	193	NS	NS	61 (7.75)	143
Phistkul et al., 2008 [55]/23	Prospective	USA	47	52	91	39	52	NS	91	NS	4	59 (7.53)	145
Hsu et al., 2010 [56]/14	Prospective	Taiwan	54	100	509	191	243	75	314	195	4	66 (8.2)	129
Baggio et al., 2002 [37]/02	Cross-sectional	Italy	58	73	96	48	48	NS	96	NS	11	65 (8.1)	NS
Cederholm et al., 2005 [57]/06	Cross-sectional	Sweden	67	59	31,037	4532	26,505	NS	4811	26,226	8	51 (6.85)	147
Savage et al., 1995 [58]/26	Cross-sectional	USA	58	61	931	264	230	439	402	531	9	103 (11.6)	NS
Okhuma et al., 2016 [59]/21	Cross-sectional	Japan	65	100	2770	760	559	1451	NS	NS	19	57 (7.40)	130
Prashanth et al., 2010 [60]/25	Cross-sectional	Oman	NS	51	447	85	362	NS	163	284	10	70 (8.55)	NS
Corradi et al., 1993 [61]/10	Cross-sectional	Italy	NS	100	90	44	46	NS	46	44	NS	60 (7.65)	162
Anan et al., 2007 [62]/01	Cross-sectional	Japan	45	18	55	20	35	NS	NS	NS	5	60 (7.65)	129
Yoem et al., 2016 [63]/31	Cross-sectional	Korea	63	100	629	314	90	225	455	174	9	58 (7.44)	126
Forsblom et al., 1998 [64]/11	Prospective^a^ (follow-up data)	Finland	58	61	108	36	54	NS	31	59	9	95 (10.8)	152
Tomlinson et al., 2006 [65]/27	Cross-sectional	China	53	100	496	196	300	NS	NS	NS	3	63(7.94)	133
Kanauchi et al., 1998 [66]/16	Cross-sectional	Japan	65	46	155	44	111	NS	78	77	13	56 (7.3)	NS
Gambaro et al., 2001 [67]/12	Prospective	Italy	65	55	273	72	134	67	107	203	13	75 (9.0)	NS
West et al., 1980 [68]/30	Cross-sectional	USA	NS	NS	973	323	421	229	416	557	7	NS	137
Klein et al., 1993 [69]/17	Cross-sectional	USA	NS	NS	376	53	200	123	58	318	NS	NS	NS
Bruno et al., 1996 [70]/04	Cross-sectional	Italy	69	43	1521	NS	NS	NS	756	765	11	64 (8.05)	NS
Bruno et al., 2003 [71]/05	Prospective	Italy	68	38	1103	149	708	222	426	677	10	65 (8.1)	154
Bentata et al., 2016 [72]/03	Prospective	Morocco	65	NS	671	81	590	NS	520	151	8	68 (8.4)	NS
Gerstein et al., 2000 [43]/13	Cross-sectional	Canada	65	63	3503	538	N/A	1777	1128	2375	11	58 (7.46)	142
Kohler et al., 2000 [73]/18	Cross-sectional	USA	51	32	1044	NS	NS	NS	244	760	0.3	76 (9.1)	NS
Nilsson et al., 2004 [74]/20	Cross-sectional	Sweden	65	54	40,648	4512	36,136	NS	5578	35,070	8	48 (6.55)	144
Parving et al., 2006 [76]/22	Cross-sectional	Denmark	61	50	24,151	NS	NS	NS	NS	NS	8	58 (7.5)	NS
Pijls et al., 2001 [75]/24	Cross-sectional	Netherlands	64	49	335	NS	NS	NS	NS	NS	6	NS	143

*N/S* not specified

^a^Both groups are normoalbuminuric at the baseline

Study-level data were also compiled for HbA1c, TC, HDL cholesterol, triglyceride, BMI, SBP and DBP. Continuous data were expressed as mean ± SD (standard deviation). For cross-sectional studies prevalence data, and for prospective studies baseline data, were extracted. In prospective studies, if albuminuria was absent at the baseline but was present at follow-up, then the follow-up data were obtained. Extracting data from all studies at just one time point, allowed both cross-sectional studies and cohort studies to be combined in the meta-analyses. The study team used the Newcastle–Ottawa Tool for the assessment of the quality of observational studies to assess the quality of included studies [12]. A random effect meta-analysis was conducted to assess the odds of having albuminuria between smokers and non-smokers. Further random effects meta-analyses models were fitted to compare participants with and without albuminuria for other risk factors (age, sex, duration of type 2 diabetes systolic and diastolic blood pressure, total cholesterol, HDL cholesterol, triglyceride, BMI and HbA1c), with categorical outcomes fitted as odds ratios and continuous variables as difference in mean values. To explore the relationship between smoking and albuminuria, meta-regression analyses were carried out. To investigate the influence of duration of diabetes on the risk of albuminuria between smokers and non-smokers further, we used the mixed effect meta-regression model to predict the odds ratio and corresponding 95% confidence intervals of albuminuria, among smokers compared to non-smokers for the duration of type 2 diabetes ranging from 4 to 20 years.

The heterogeneity between studies was assessed using the *I*^2^ statistic, which represents the total proportion of study variation that is due to heterogeneity rather than sampling error/chance [13]. Publication bias among studies was assessed by visual inspection of the funnel plot and the Egger’s test. The type 1 error to determine the level of statistical significance was set at *p* = 0.05. All statistical analyses were carried out using the metafor package (version 2.0.0) in the R statistical software environment and Cochrane Collaboration Review Manager version 5.

## Results

**Fig. 2:**
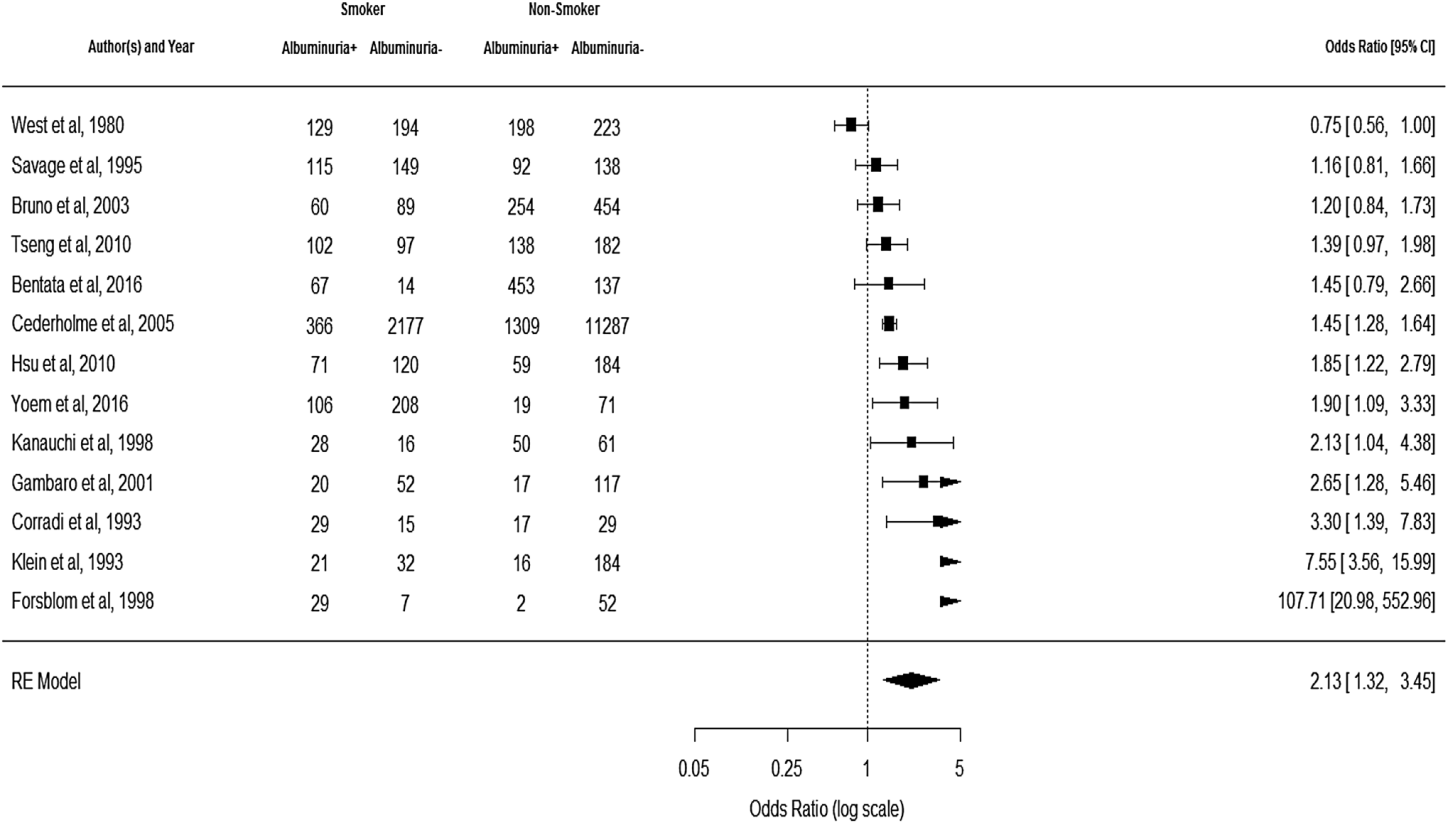
Forest plot showing an odds ratio of albuminuria in smokers compared to non-smokers

A total of 2207 studies were identified by electronic database searches. After removing the duplicates, 2119 articles were screened for eligibility; 150 of them were accepted for abstract review, and 58 of them were included for full-text review. Overall, 30 studies (20 cross-sectional and ten prospective observational) with a total of 113,140 people with T2DM were included. The mean age of the study participants was 58 years, and 51% of them were male. Amongst the study participants, 11% were smokers, 60% were non-smokers, and 4% were quitters. Smoking status was unavailable for 25% of the study participants. The prevalence of albuminuria in the included studies was 14%. The mean duration of T2DM was 8 years; the mean HbA1c was 63 mmol/mol (7.9%), and the mean SBP was 125 mmHg. The outcomes from the random effects meta-analysis of 13 studies on 4313 smokers and 15,743 non-smokers showed that the pooled odds ratio of albuminuria in smokers, compared to non-smokers was 2.13 (95% CI 1.32–3.45; *p* = 0.002; Fig. 2), indicating a statistically significant increased risk of albuminuria in smokers.

**Fig. 3:**
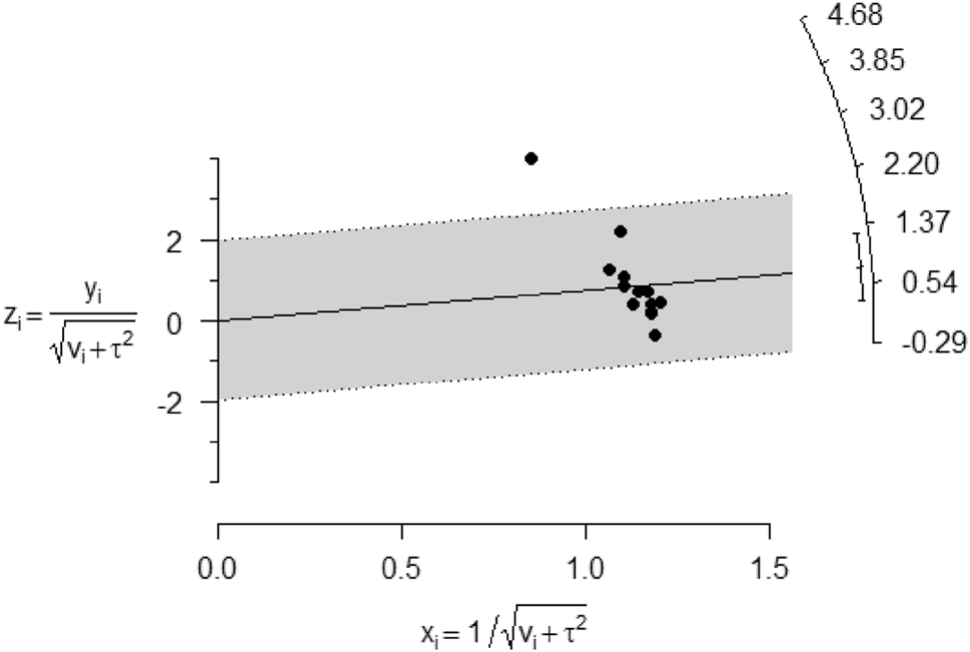
A radial plot of random effects meta-analysis showing the standardized differences in observed outcomes (zi) between smokers against their corresponding precision (xi). The plot demonstrates that the differences in outcomes between smokers and non-smokers were consistent for most studies suggesting that other factors were unlikely to contribute to the variation in the risk of albuminuria

Except for one study, the radial plot suggested that the outcomes for most of the studies were consistent, regarding the effects of smoking and the variation in the risk of albuminuria (Fig. 3).

The visual exploration of the funnel plot showed slight asymmetry of the plot suggesting possible publication bias. However, outcomes from the Egger’s test showed the evidence was not statistically significant (*p* = 0.063) (Supplementary material 2).

Further meta-analyses demonstrated that cardiometabolic factors associated with albuminuria (OR, 95% CI) were: age 1.24 (95% CI 0.84–1.64, *p* < 0.001); male sex 1.39 (95% CI 1.16–1.67; *p* = 0.003); SBP 6.03 (95% CI 4.10–7.97, *p* < 0.001); DBP 1.85 (95% CI 1.08–2.62, *p* < 0.001); duration of T2DM 1.78 (95% CI 1.32–2.23, *p* < 0.001); BMI 0.40 (95% CI 0.00–0.80, *p* = 0.05); total cholesterol 0.06 (95% CI − 0.05 to 0.17; *p* = 0.31); HDL − 0.01 (95% CI − 0.04 to 0.02; *p* = 0.47); triglyceride 0.22 (95% CI 0.12–0.33; *p* < 0.001) and HbA1c 0.63 (95% CI 0.45–0.81; *p* < 0.001) (Table 2) (Supplementary material 3).

**Table 2:** Relationship of cardiometabolic risk factors and albuminuria before adjusting for smoking status

Variables	Mean difference	95% confidence interval	*p* value
Age	1.24	0.84–1.64	< 0.001
Male sex	1.39	1.16–1.67	0.003
SBP	6.03	4.10–7.97	< 0.001
DBP	1.85	1.08–2.62	< 0.001
HbA1c	0.63	0.45–0.81	< 0.001
Duration of diabetes	1.78	1.32–2.23	< 0.001
Total cholesterol	0.06	− 0.05 to 0.17	0.31
HDL cholesterol	− 0.01	− 0.04 to 0.02	0.47
Triglyceride	0.22	0.12–0.33	< 0.001
Body mass index	0.40	− 0.00 to 0.80	0.05

Meta-regression analyses found most moderator variables were not associated with the study effect except the duration of diabetes showing a significant association (*p* = 0.001). We observed that the inclusion of duration of diabetes as a moderator variable reduced the residual heterogeneity although there was still evidence of residual heterogeneity (*Q* statistic = 10.09, *p* = 0.002); the estimate of residual heterogeneity (*τ*^2^) reduced from 0.69 (95% CI 0.38–3.84) based on the random effect meta-analysis model to 0.23 (95% CI 0.10–2.13) based on the mixed effect meta-regression model (Table 3) (Supplementary material 3). Therefore, the time to diabetes as a moderator variable accounted for almost 60% of the heterogeneity.

**Table 3:** Relationship of cardiometabolic risk factors with albuminuria after adjusting for smoking status

Moderator variables	Overall effect size (*Z*)	Heterogeneity (*τ*^2^)	*p* value
Age	0.75 (− 0.084–0.18)	0.70 (0.33–6.44)	0.46
Male sex	0.27 (− 0.02–0.03)	0.79 (0.36–6.81)	0.78
HbA1c	1.43 (0.1–0.65)	0.76 (0.30–4.94)	0.15
HDL	− 0.50 (− 47.78 to 28.83)	9.93 (1.66–100)	0.61
Total cholesterol	0.92 (− 1.36 to 3.75)	1.74 (0.56–15.78)	0.35
Triglyceride	− 1.14 (− 0.51 to 0.14)	0.01 (0–1.28)	0.25
**Duration of diabetes**	**3.18 (0.07–0.31)**	**0.23 (0.10–2.13)**	**0.001**
SBP	1.09 (− 0.29 to 0.101)	1.26 (0.44–10.22)	0.27
DBP	0.26 (− 0.13 to 0.17)	2.05 (0.66–18.43)	0.79
BMI	2.48 (0.15–1.30)	0.74 (0.36–6.86)	0.93

Statistically significant variable that influenced the relationship between smoking and albuminuria was the duration of T2DM (highlighted in bold font)

**Fig. 4:**
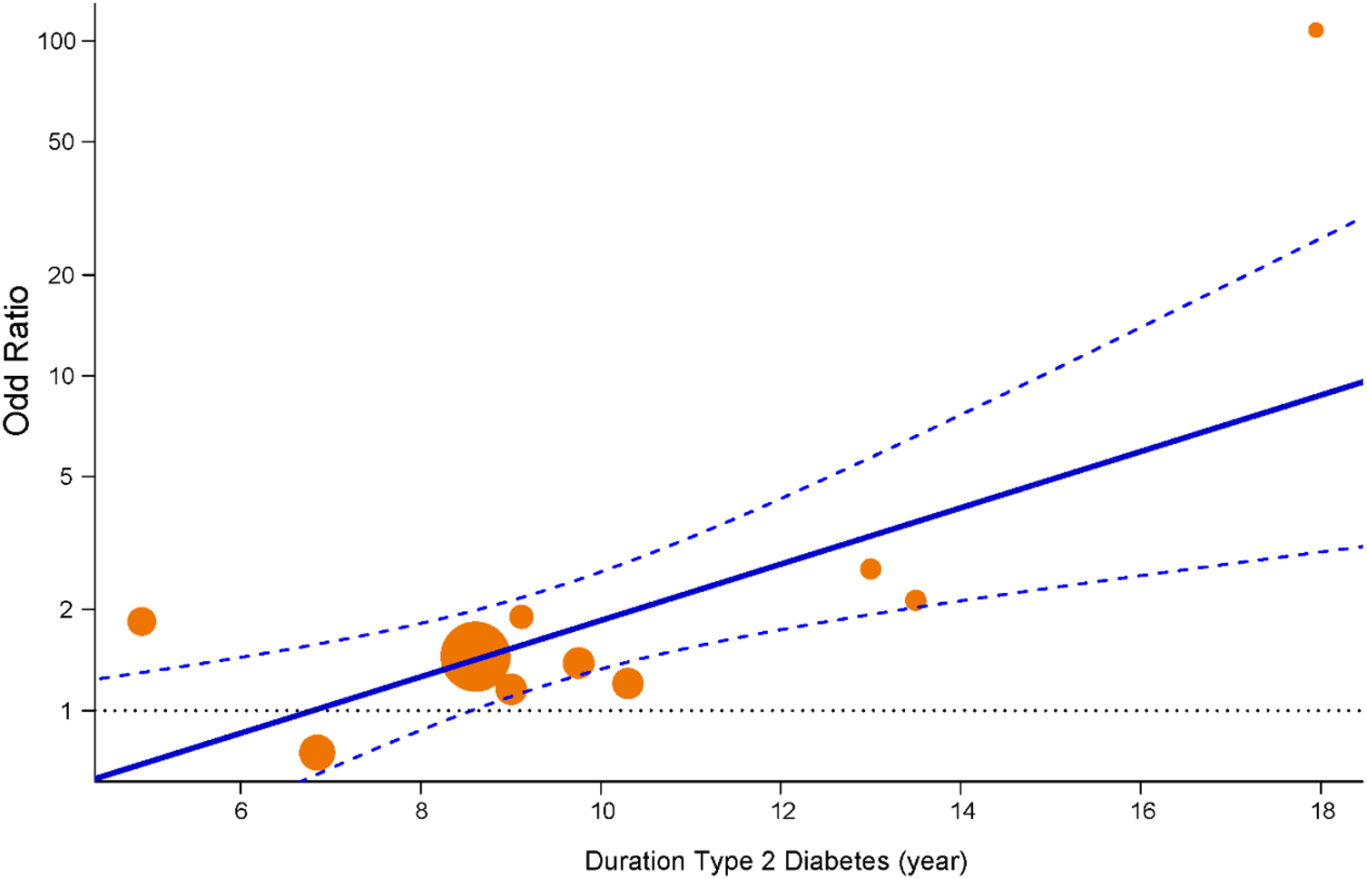
Predicted odds ratio (OR) of albuminuria in smokers compared to non-smokers with duration of type 2 diabetes based on the outcome of the logistic mixed model. The solid line shows the predicted mean and dashed line shows the corresponding 95% confidence interval. The OR below the horizontal dotted line is not statistically significant (*p* > 0.05). The plot also shows the observed OR of individual studies (points) where the point sizes are proportional to the inverse of the corresponding standard errors

The statistically significant residual heterogeneity suggested that other moderators not investigated in this study might be important. The duration of T2DM was positively associated with albuminuria: each year increase in the duration of T2DM was associated with an increased log of odds of albuminuria on an average by 0.19 units (95% CI 0.07–0.31), or it increased the odds approximately by 21% (Fig. 4). After 9 years of diabetes, the odds of albuminuria in smokers was approximately 50% higher 1.53 (1.10–2.43) compared to non-smokers. The odds ratio rose further to almost three times at 12-year duration 2.74 (1.74–4.30) and almost six times after 16 years 5.94 (2.58–15.05). The predicted mean odds ratio of albuminuria among smokers compared to non-smokers conditional on a range of the duration of diabetes are presented in Supplementary material 4.

## Discussion

This systematic review summarises the relationship between smoking and albuminuria in people with T2DM, and whether this relationship is influenced by other confounding variables such as age, sex, the duration of T2DM, HbA1c, BMI, HDL and total cholesterol, systolic and diastolic blood pressure. The meta-analysis suggests that smoking is a strong predictor of albuminuria in people with T2DM. The meta-regression, on the other hand, concedes that apart from the duration of T2DM, none of the above confounding variables has any statistically significant influence on albuminuria, when adjusted for smoking status. There is a linear relationship between smoking and the duration of T2DM with albuminuria. Smokers with T2DM have 21% increased annual risk of albuminuria, compared to non-smokers. Therefore, smoking cessation at an early stage of disease trajectory is likely to be one of the most effective intervention strategies to prevent the development of albuminuria in smokers with T2DM.

This is the first systematic review and meta-analysis exploring the relationship between smoking and albuminuria, and how other cardiometabolic parameters influence this relationship. Although multiple studies have shown smoking augments the risk of albuminuria in people with type 1 diabetes [14, 15], its role in T2DM remains undetermined. T2DM, as opposed to T1DM, is one of the components of metabolic syndrome. In addition to hyperglycaemia, it is often accompanied by obesity, hypertension and dyslipidaemia [16]. All these risk factors are closely associated with albuminuria [17], and therefore, the relationship between smoking and albuminuria is much more intricate in T2DM, compared to T1DM. Previous studies have shown that smokers have a higher urinary albumin excretion rate, which might have been independent of glycaemic effects [18]. Meta-analyses in this systematic review concluded that there is a close association between smoking and albuminuria in people with T2DM. Meta-regression, on the other hand, taking into consideration all the above confounding variables, concluded that the duration of diabetes is the most important predictor of albuminuria in smokers with T2DM.

Early detection of albuminuria at the stage of microalbuminuria, and multifactorial intervention including smoking cessation, are advocated in all the guidelines across the globe, including the European Association of Study on Diabetes (EASD) and the American Diabetes Association (ADA) [19]. This recommendation is based on the observation that once the daily urinary albumin excretion rate reaches the level of proteinuria (urinary albumin excretion > 300 mg/day), no interventions appears to be effective in reversing it [20, 21]. Addressing other anthropometric and metabolic risk factors including hip–waist ratio, BMI, HbA1c, blood pressure and lipid profiles remain at the centre of this intervention strategy. For glycaemic management, the choice of drugs seems to be a determinant factor of albuminuria. Insulin sensitizers have shown better efficacy in halting the prevalence and progression of albuminuria compared to insulin and its secretagogues. In BARI-2D trial, the researchers have shown that insulin, and its secretagogues are more likely to cause an increased prevalence of albuminuria and coronary artery disease, compared to insulin-sensitizing drugs [22]. However, it will be interesting to know if this outcome is influenced by the choice of drugs or people who were on insulin had poorer glycaemic control.

Irrespective of hypertension, treatment with angiotensin-converting enzyme inhibitors (ACEI) or angiotensin receptor blockers (ARB) has shown promising prospect of halting the prevalence and progression of albuminuria [23]. However, studies have shown that this reno-protective effect of ACEI and ARBs can be revoked in smokers [24], suggesting that renin–angiotensin axis blockade is less effective to prevent the progression of albuminuria in smokers. Raised triglyceride, and raised total and LDL cholesterol, with low HDL cholesterol are the hallmarks of dyslipidaemia in T2DM [25], but in smokers the predominant abnormality in lipid profile seems to be lower HDL cholesterol [26]. Smoking downregulates the hepatic and endothelial lipoprotein lipase activities [27] and tampers with the reverse cholesterol transport pathway [28]. As a consequence, they have lower HDL cholesterol compared to their non-smoker counterparts. Smoking cessation, on the other hand improves lipid profile particularly the HDL cholesterol, despite moderate weight gain [29, 30], which in turn halts the progression of albuminuria [31]. Conversely, isolated and piecemeal management of glucose, blood pressure and lipid profile did not show consistent efficacy to prevent the prevalence or progression of albuminuria in smokers with diabetes [9, 32]. These observations are suggestive of an independent relationship between smoking and albuminuria mediated by a constellation of underlying pathophysiological processes.

Several mechanisms have been proposed to explain the albuminuria in smokers with T2DM. They include increased blood pressure, altered intrarenal haemodynamics such as activation of the sympatho-adrenergic pathway, activation of the renin–angiotensin–aldosterone axis and the endothelin system [33–35]. In addition, smoking directly causes tubulo-interstitial disease [36] and causes neuro-endocrine disruption, vascular endothelial damage and metabolic deregulations which adversely affect renal structure and function [18, 37, 38]. Therefore, addressing hyperglycaemia, hypertension and dyslipidaemia without smoking cessation may not halt the prevalence and progression of albuminuria in smokers with diabetes.

Nicotine and other toxic metabolites in cigarettes appear to be handled differently in people with and without diabetes [39]. Nicotine infusion acutely increases insulin resistance in people with T2DM but not in people without [40]. Although smokers have lower BMI than non-smokers, nonetheless they have more visceral adiposity and lower insulin sensitivity [41]. Smoking cessation, on the other hand, despite causing moderate weight gain, is associated with the reversal of visceral adiposity and an improvement in insulin sensitivity [30]. But this reversal takes longer in people with T2DM, compared to people without [3, 42]. Therefore, short-term abstinence may not yield any meaningful benefit in smokers with T2DM. The Heart Outcomes Prevention Evaluation (HOPE) study examined the factors that influence the prevalence and progression of albuminuria in people with and without diabetes. This study demonstrated that smoking, hypertension, older age, abdominal adiposity, vascular disease and left ventricular hypertrophy were significantly associated with albuminuria, in people with and without diabetes. However, in people with diabetes, the most significant determinants of albuminuria were the duration of diabetes, HbA1c and the use of insulin. People with diabetes were 1.16 times more likely to develop albuminuria after a diabetes duration of 10.4 years (irrespective of their HbA1c), the risk of albuminuria increased by 8% for each 0.9% increase in the HbA1c, and the people with albuminuria were 1.3 times more likely to be on insulin compared to people who had normoalbuminuria. Sex, dyslipidaemia, creatinine, and BMI were not independently associated with albuminuria after adjustment of other factors [43]. Taking all these evidence into account, this systematic review emphasises that to effectively manage the prevalence and the progression of albuminuria in T2DM, the most effective strategy would be a multifactorial intervention where smoking cessation is one of the key components.

The findings of this systematic review have significant clinical implications. The World Health Organization (WHO) estimates that by 2030, a staggering number of 366 million people will suffer from T2DM worldwide. Amongst them, 60 million will be between 20 and 44 years, and 180 million will be between 45 and 64 years [44]. Young smokers with T2DM are at a higher risk of albuminuria as they will live longer with the condition. This study showed that the risk of albuminuria was similar in smokers and non-smokers up to around 8.5 years of T2DM duration, and then the risk increased approximately by 20% annually. Albuminuria marks the onset of microvascular complications which is often associated with retinopathy, neuropathy and macrovascular involvement [45]. Several studies have shown a rapid rise in the prevalence of albuminuria and cardiovascular complications in younger patients with T2DM, compared to T1DM, despite having similar glycaemic control [46, 47]. Poor lifestyle choices including smoking have been attributed to this disparate response of glycaemic control in T2DM, as opposed to T1DM. Therefore, this study emphasises that smokers, particularly the younger smokers should be encouraged to quit soon after the diagnosis and persuaded to remain abstinent.

One of the strengths of this study is that it included all the major studies available on the electronic databases from their inception and included 30 studies with 113,400 participants. The quality of the papers was determined by the Newcastle–Ottawa scale, which is a validated tool, and the review process followed PRISMA protocol [10], which is considered to be the gold standard. Publication bias was addressed by conducting the appropriate sensitivity test, which did not show any significant bias. On the other hand, the weaknesses of the study were that it was based mainly on cross-sectional, or the baseline data of prospective studies, and therefore, no temporal relationship between smoking and albuminuria can be confirmed. Second, most of the included studies used self-reported smoking behaviour which might not be accurate. There was also considerable heterogeneity in the included studies, and therefore, the findings may not be generalisable. Although between-study heterogeneity was investigated, meta-regression models lacked statistical power to assess associations between the effect size and study-level covariates.

## Conclusion

Albuminuria is one of the earliest biochemically measurable risk factors in T2DM, which heralds incipient micro- and macrovascular complications. It is a substantial milestone in the trajectory of disease progression and is independently associated with cardiovascular and all-cause mortality. This study reiterates that smoking is a strong predictor of albuminuria; the longer the duration of T2DM, the higher the risk. With a rapidly changing global prevalence of T2DM with an anticipated surge of younger people with T2DM [48], and an approximately 70% of them already having complications [49], it is important to raise awareness about the effect of smoking and duration of T2DM on albuminuria, and its impact on cardiovascular mortality. Future research should be focused on elucidating the relationship between smoking cessation, and the progression of albuminuria in people with T2DM, particularly the length of abstinence required to reverse the risk of albuminuria.

## Electronic supplementary material

Below is the link to the electronic supplementary material.

Supplementary material 1 (DOCX 45 KB)

Supplementary material 2 (DOCX 206 KB)

Supplementary material 3 (DOCX 1190 KB)

## Abbreviations

T1DM: Type 1 diabetes mellitus
T2DM: Type 2 diabetes mellitus
HbA1c: Glycosylated haemoglobin
HDL: High-density lipoprotein
LDL: Low-density lipoprotein
SBP: Systolic blood pressure
DBP: Diastolic blood pressure
