# Supplementary material for: Association of smoking and cardiometabolic parameters with albuminuria in people with type 2 diabetes mellitus: a systematic review and meta-analysis
Source: Acta Diabetol. 2019 Feb 24;56(8):839–50. doi: 10.1007/s00592-019-01293-x (PMC6597612; doi:10.1007/s00592-019-01293-x)
Supplement: Supplementary file 1 — Supplementary material 1 (DOCX 45 KB) [file 592_2019_1293_MOESM1_ESM.docx]

## SUPPLEMENTARY MATERIAL 1

## Search strategy

1. **Medline**

Database: Ovid MEDLINE(R) Epub Ahead of Print, In-Process & Other Non-Indexed Citations, Ovid MEDLINE(R) Daily and Ovid MEDLINE(R) <1946 to Present>

Search Strategy:

--------------------------------------------------------------------------------

1 diabetes.mp. or exp Diabetes Mellitus/ (574423)

2 exp type 2 diabetes mellitus/ (114464)

3 T2DM.mp. (14940)

4 (NIDDM or non insulin dependent diabetes mellitus or maturity onset diabetes mellitus).mp. [mp=title, abstract, original title, name of substance word, subject heading word, keyword heading word, protocol supplementary concept word, rare disease supplementary concept word, unique identifier, synonyms] (10171)

5 exp type 1 diabetes mellitus/ (70406)

6 T1DM.mp. (3574)

7 (IDDM or insulin dependent diabetes mellitus or juvenile diabetes mellitus).mp. [mp=title, abstract, original title, name of substance word, subject heading word, keyword heading word, protocol supplementary concept word, rare disease supplementary concept word, unique identifier, synonyms] (18607)

8 Diabetes Mellitus, Type 2/ (114279)

9 steroid induced diabetes.mp. (125)

10 exp Diabetes Insipidus/ (7588)

11 1 or 2 or 3 or 4 or 8 (574726)

12 5 or 6 or 7 (79984)

13 11 not 12 (494858)

14 9 or 10 (7713)

15 13 not 14 (487617)

16 (non-smok* or nev* smo*).mp. [mp=title, abstract, original title, name of substance word, subject heading word, keyword heading word, protocol supplementary concept word, rare disease supplementary concept word, unique identifier, synonyms] (27248)

17 smok*.mp. or exp "Tobacco Use Disorder"/ (295834)

18 (quit* or abst* or giv* up*).mp. [mp=title, abstract, original title, name of substance word, subject heading word, keyword heading word, protocol supplementary concept word, rare disease supplementary concept word, unique identifier, synonyms] (1999011)

19 (ACR* or microalb* or alb* creat* rati*).mp. [mp=title, abstract, original title, name of substance word, subject heading word, keyword heading word, protocol supplementary concept word, rare disease supplementary concept word, unique identifier, synonyms] (692935)

20 (macroalb* or proteinur*).mp. [mp=title, abstract, original title, name of substance word, subject heading word, keyword heading word, protocol supplementary concept word, rare disease supplementary concept word, unique identifier, synonyms] (47235)

21 (ex-smok* or form* smok* or quit*).mp. [mp=title, abstract, original title, name of substance word, subject heading word, keyword heading word, protocol supplementary concept word, rare disease supplementary concept word, unique identifier, synonyms] (128035)

22 19 not 20 (689572)

23 16 or 17 or 18 or 21 (2253456)

24 (Prevalen* or Progres*).mp. [mp=title, abstract, original title, name of substance word, subject heading word, keyword heading word, protocol supplementary concept word, rare disease supplementary concept word, unique identifier, synonyms] (1710008)

25 22 and 24 (64833)

26 23 and 25 (6256)

27 15 and 26 (926)

**2. Embase**

1 EMBASE(diabetes mellitus).ti,ab 230242

2 EMBASE (Prediabetic state).ti,ab 414

3 EMBASE (metabolic syndrome X).ti,ab 220

4 EMBASE *"DISORDERS OF CARBOHYDRATE METABOLISM"/ 1589

5 EMBASE "GLUCOSE INTOLERANCE"/ 15639

6 EMBASE HYPERGLYCEMIA/ OR "HYPERGLYCEMIC SYNDROME"/ 83334

7 EMBASE (glucose metabolism disorder).ti,ab 102

8 EMBASE exp "INSULIN SENSITIVITY"/ OR exp "INSULIN SENSITIVITY TEST"/ OR exp "INSULIN RESISTANCE"/ OR exp "INSULIN RESISTANCE SYNDROME"/ 180423

9 EMBASE exp "IMPAIRED GLUCOSE TOLERANCE,POTENTIAL"/ 25822

10 EMBASE exp "NON INSULIN DEPENDENT DIABETES MELLITUS"/ 207452

11 EMBASE exp "CIGARETTE SMOKING"/ OR exp "CIGARETTE SMOKER"/ OR exp "CIGARETTE SMOKE"/ 300980

12 EMBASE exp "SMOKING HABIT"/ 20073

13 EMBASE exp "SMOKING CESSATION"/ 50896

14 EMBASE exp "SMOKELESS TOBACCO"/ 4200

15 EMBASE (tobacco use disorder).ti,ab 160

16 EMBASE exp "PASSIVE SMOKING"/ 10926

17 EMBASE exp SMOKING/ 293853

18 EMBASE exp "BIDI SMOKING"/ 111

19 EMBASE (smoking AND related phenomena).ti,ab 7

20 EMBASE (quit OR give up OR cease).ti,ab 23980

21 EMBASE exp MICROALBUMINURIA/ OR exp ALBUMINURIA/ 27379

22 EMBASE exp "HEMOGLOBIN A1C"/ OR exp "GLYCOSYLATED HEMOGLOBIN"/ 98487

23 EMBASE exp AGE/ 814644

24 EMBASE exp "BLOOD PRESSURE"/ 511841

25 EMBASE exp OBESITY/ 433634

26 EMBASE (Body mass index OR BMI).ti,ab 348257

27 EMBASE (Cholesterol OR lipid OR high density lipoprotein OR low density lipoprotein OR triglyceride).ti,ab 673528

28 EMBASE exp CHOLESTEROL/ 280274

29 EMBASE exp "INSULIN DEPENDENT DIABETES MELLITUS"/ OR exp "LATENT AUTOIMMUNE DIABETES IN ADULTS"/ 100682

30 EMBASE exp "DIABETES INSIPIDUS"/ 12977

31 EMBASE (1 OR 2 OR 3 OR 4 OR 5 OR 6 OR 7 OR 8 OR 9 OR 10) 560487

32 EMBASE (29 OR 30) 113388

33 EMBASE 31 NOT 32 507140

34 EMBASE (11 OR 12 OR 13 OR 14 OR 15 OR 16 OR 17 OR 18 OR 19 OR 20) 340408

35 EMBASE (21 AND 34) 2022

36 EMBASE (33 AND 34 AND 35) 935
