# Supplementary material for: Association of smoking and cardiometabolic parameters with albuminuria in people with type 2 diabetes mellitus: a systematic review and meta-analysis
Source: Acta Diabetol. 2019 Feb 24;56(8):839–50. doi: 10.1007/s00592-019-01293-x (PMC6597612; doi:10.1007/s00592-019-01293-x)

## SUPPLEMENTARY MATERIAL 2 (Meta-analyses 1)

1. **Data**

**Supplementary table 1 – Cardiometabolic parameters in smokers vs. non-smokers (13 studies)**

| ID | Author | Year | Design | Country | Male (%) | Participants  (n) | Age | SBP | DBP | HbA1c | DM duration | HDL | BMI | TG | TC | Alb+ve Smoker | Alb-ve  Smoker | Alb+ve non-smoker | Alb-ve non-smoker |  |  |
| --- | --- | --- | --- | --- | --- | --- | --- | --- | --- | --- | --- | --- | --- | --- | --- | --- | --- | --- | --- | --- | --- |
| S03 | Bentata | 2016 | Prospective | Morocco | NS | 671 | 65.00 | NS | NS | 8.3 | 8.5 | NS | 27.56 | 1.53 | NS | 67 | 14 | 453 | 137 |  |  |
| S05 | Bruno | 2003 | Prospective | Italy | 38 | 1103 | 67.50 | 154.26 | 87.50 | 8.03 | 10.30 | 1.44 | 27.55 | NS | 5.81 | 60 | 89 | 254 | 454 |  |  |
| S06 | Cederholme | 2005 | Cross-sectional | Sweden | 59 | 15139 | 66.56 | 143.82 | 78.37 | 6.61 | 8.61 | NS | 28.82 | 1.86 | 5.17 | 366 | 2177 | 1309 | 11287 |  |  |
| S10 | Corradi | 1993 | Cross-sectional | Italy | 100 | 90 | NS | 161.71 | 101.76 | 7.65 | NS | NS | NS | NS | NS | 29 | 15 | 17 | 29 |  |  |
| S11 | Forsblom | 1998 | Prospective | Finland | 61 | 108 | 66.00 | 156.69 | 86.31 | 11.65 | 17.95 | 1.25 | 28.14 | NS | 5.92 | 29 | 7 | 2 | 52 |  |  |
| S12 | Gambaro | 2001 | Prospective | Italy | 55 | 273 | 65.00 | NS | NS | 9.00 | 13.00 | NS | 27.95 | 1.88 | 6.09 | 20 | 52 | 17 | 117 |  |  |
| S14 | Hsu | 2010 | Prospective | Taiwan | 100 | 509 | 54.70 | 128.96 | 81.38 | 8.13 | 4.90 | NS | 25.76 | 2.06 | 4.91 | 71 | 120 | 59 | 184 |  |  |
| S16 | Kanauchi | 1998 | Cross-sectional | Japan | 46 | 155 | 65.00 | NS | NS | 7.26 | 13.50 | NS | 22.96 | NS | NS | 28 | 16 | 50 | 61 |  |  |
| S17 | Klein | 1993 | Cross-sectional | USA | NS | 376 | NS | NS | NS | NS | NS | NS | NS | NS | NS | 21 | 32 | 16 | 184 |  |  |
| S26 | Savage | 1995 | Cross-sectional | USA | 61 | 931 | 58.28 | 146.52 | 91.29 | 11.64 | 9.00 | NS | 30.50 | 3.26 | 5.70 | 115 | 149 | 92 | 138 |  |  |
| S28 | Tseng | 2010 | Prospective | Taiwan | 55.3 | 519 | 58.43 | 132.34 | 82.87 | 7.98 | 9.75 | 1.24 | 24.88 | NS | 5.38 | 102 | 97 | 138 | 182 |  |  |
| S30 | West | 1980 | Prospective | USA | NS | 973 | NS | NS | NS | NS | 6.85 | NS | NS | NS | NS | 129 | 194 | 198 | 223 |  |  |
| S31 | Yoem | 2016 | Cross-sectional | Korea | 100 | 629 | 62.51 | 126.09 | NS | NS | 9.12 | NS | 24.30 | NS | NS | 106 | 208 | 19 | 71 |  |  |

1. **Model – Random effect meta-analysis model without moderator**

Meta-analysis regression was conducted accounting for important moderator variables recorded in these studies. First, a random effect model was fitted to assess the risk of developing Albuminuria for the smoker and non-smoker groups.

**Abbreviations**

- Alb+ve – Albuminuria positive
- Alb-ve – Albuminuria negative
- NS – not specified
- CI – confidence interval
- LB – Lower boundary
- UB – upper boundary

1. **Parameters**

- tau^2: Estimated amount of residual heterogeneity
- tau: square root of estimated tau^2 value
- I^2: residual heterogeneity / unaccounted variability
- H^2: unaccounted variability / sampling variability
- R^2: amount of heterogeneity accounted for
- QE: Statistic to test for Residual Heterogeneity
- QM: Statistic to test Moderators

1. **Model outputs**

Total number of studies 13 (Supplementary table 1)

| Parameters | Estimate | ci.lb | ci.ub |
| --- | --- | --- | --- |
| Tau^2 | 0.6851 | 0.3783 | 3.8372 |
| Tau | 0.8277 | 0.6150 | 1.9589 |
| I^2(%) | 94.7179 | 90.6594 | 98.9946 |
| H^2 | 18.5801 | 10.7060 | 99.4634 |

QE = 76.59636; p-value = 1.832091e-11

|  | estimate | se | z-val | p-val | ci.lb | ci.ub |
| --- | --- | --- | --- | --- | --- | --- |
| intrcept | 0.7569 | 0.2462 | 3.0741 | 0.0021 | 0.2743 | 1.2395 |

1. **Test for funnel plot asymmetry**

Regression Test for Funnel Plot Asymmetry

Model: weighted regression with multiplicative dispersion

predictor: standard error

test for funnel plot asymmetry: t = 2.0677, df = 11, p = 0.0630


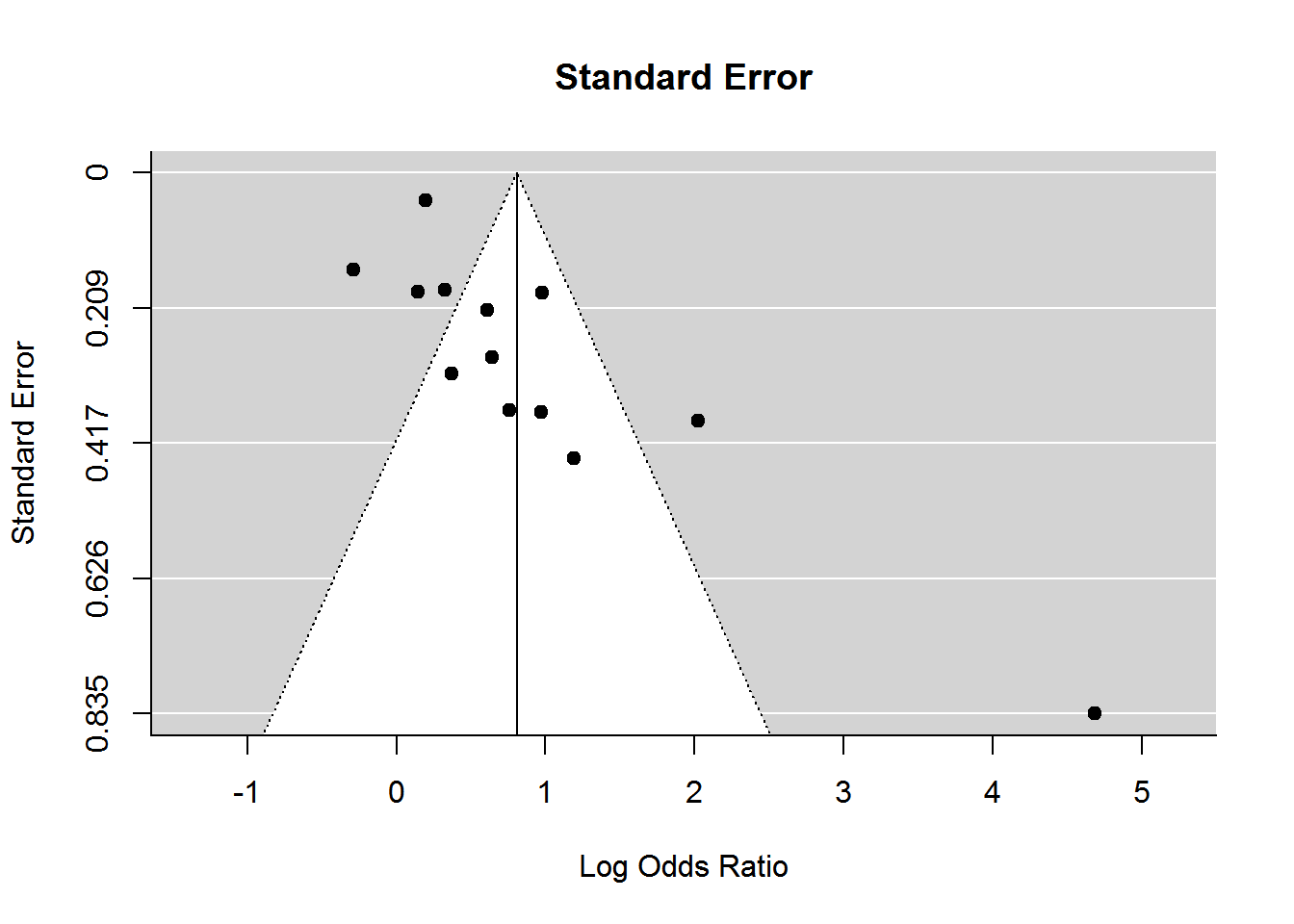


**Figure 2: Funnel plot exploring publication bias**

1. **Summary**

We fitted different mixed effect meta-regression models to assess if the residual heterogeneity is influenced by different moderator variables.

The following sections include the model outcomes from the Random Effect Meta-Regression model without Moderator and Mixed Effect Meta-Regression models with different Moderators.

The results from the meta-regression output suggest that the risk of Albuminuria positive in the smoker is significantly higher; the risk if almost 2.2 times higher in the smoker group compared with the non-smoker group.

The moderator variables explored were: age, male sex, SBP, DBP, HbA1c, duration of T2DM, HDL, BMI, triglyceride and total cholesterol.

The results did not suggest that the risk of albuminuria was associated with any of the above moderator variables except the duration of type 2 diabetes when the model was adjusted for smoking status. For the variable of the duration of T2DM, each year increase in duration of T2DM increased the likelihood of albuminuria by 1.2 times (95% confidence interval: 1.09, 1.38).

We carried out meta-analyses to explore the relationship between cardiometabolic parameters. For continuous data, random effect model with inverse variance was used. The outcome measure was expressed in mean difference with 95% confidence interval. For dichotomous data, the data was analysed using random effect, and Mantel-Haenszel method and the outcome was expressed in odds ratio with 95% confidence interval. For continuous data, an inverse variance and random effects model was fitted.

**Age and albuminuria**


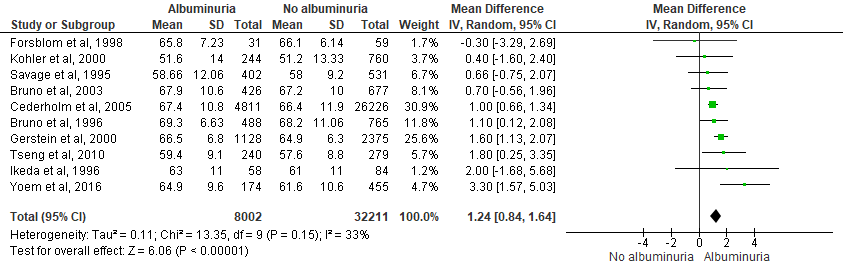


**Male sex and albuminuria**

**
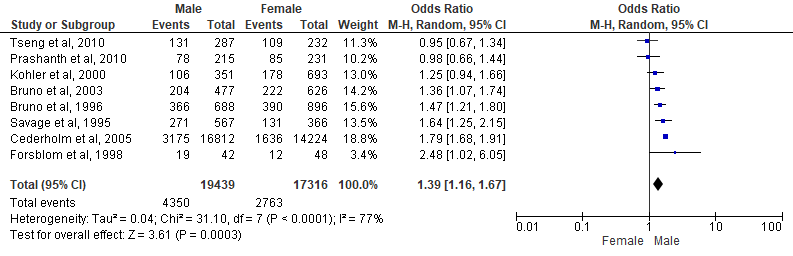
**

**Systolic blood pressure (SBP) and albuminuria**

**
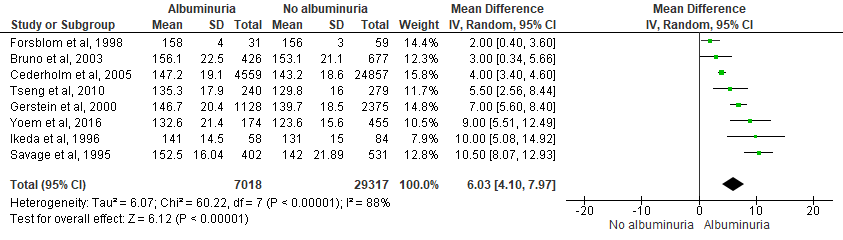
**

**Diastolic blood pressure (DBP) and albuminuria**

**
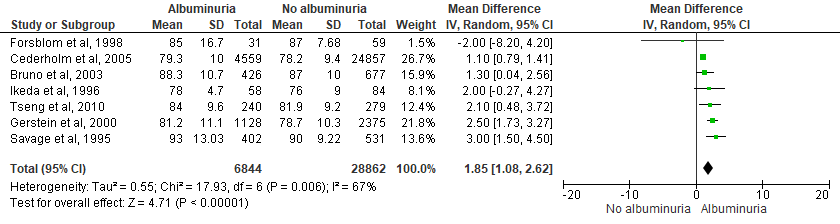
**

**Glycosylated haemoglobin (HbA1c) and albuminuria**

**
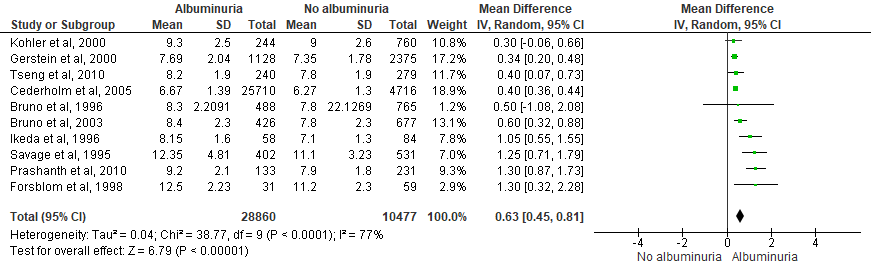
**

**Body mass index (BMI) and albuminuria**

**
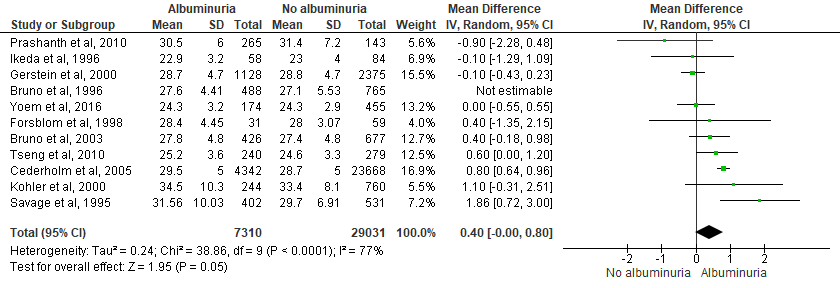
**

**Total cholesterol (TC) and albuminuria**

**
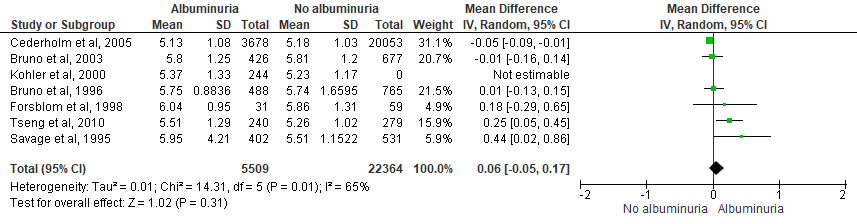
**

**Triglyceride and albuminuria**

**
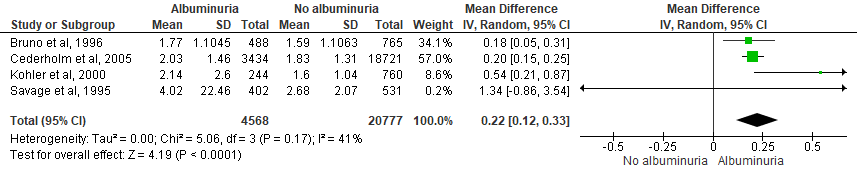
**

**High-density lipoprotein (HDL) cholesterol and albuminuria**

**
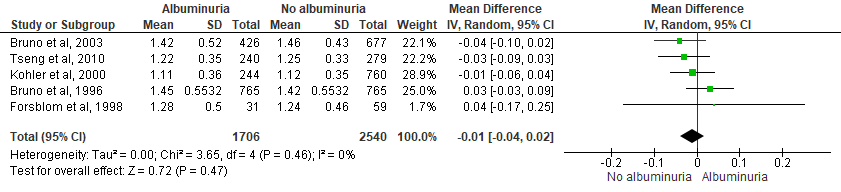
**

**Duration of type 2 diabetes mellitus and albuminuria**

##
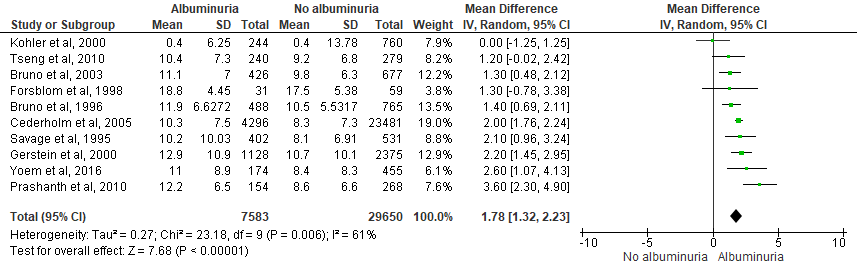

Supplement: Supplementary file 2 — Supplementary material 2 (DOCX 206 KB) [file 592_2019_1293_MOESM2_ESM.docx]
