# Supplementary material for: Association of smoking and cardiometabolic parameters with albuminuria in people with type 2 diabetes mellitus: a systematic review and meta-analysis
Source: Acta Diabetol. 2019 Feb 24;56(8):839–50. doi: 10.1007/s00592-019-01293-x (PMC6597612; doi:10.1007/s00592-019-01293-x)

## SUPPLEMENTARY MATERIAL 3 (Meta-regression)

We adjusted for different moderator variables using a mixed effects model to assess if the estimated residual heterogeneity could be explained due to the study-level moderator variables. The moderator variables explored were: Age, male sex, SBP, DBP, HbA1c, DM duration, HDL, BMI, Triglyceride (TG) and Total Cholesterol (TC). The numeric moderator variables were fitted as a deviation from the mean of the variable from all studies.

**Moderator – Age (Number of studies – 10)**

|  | estimate | ci.lb | ci.ub |
| --- | --- | --- | --- |
| tau^2 | 0.6993 | 0.3300 | 6.4420 |
| tau | 0.8362 | 0.5744 | 2.5381 |
| I^2 (%) | 93.6658 | 87.4658 | 99.2713 |
| H^2 | 15.7873 | 7.9792 | 137.2281 |

**R^2 = NA;**

**QE = 34.9842; p-val = 2.691618e-05;**

**QM = 0.526; p-val = 0.4682846**

|  | estimate | Se | z-val | p-val | ci.lb | ci.ub |
| --- | --- | --- | --- | --- | --- | --- |
| intercept | 0.5582 | 0.3575 | 1.5612 | 0.1185 | -0.1425 | 1.2589 |
| I (Age – 59.47) | 0.0493 | 0.0679 | 0.7253 | 0.4683 | -0.0839 | 0.1824 |

**Moderator - Male sex (Number of studies 10)**

|  | estimate | ci.lb | ci.ub |
| --- | --- | --- | --- |
| tau^2 | *0.7758* | *0.3567* | *6.8107* |
| tau | *0.8808* | *0.5973* | *2.6097* |
| I^2 (%) | *95.2294* | *90.1755* | *99.4326* |
| H^2 | *20.9617* | *10.1786* | *176.2466* |

***R^2 = 0***

***QE = 34.2245; p-val = 3.699244e-05;***

***QM = 0.0751; p-val = 0.7839817***

|  | estimate | Se | z-val | p-val | ci.lb | ci.ub |
| --- | --- | --- | --- | --- | --- | --- |
| intercept | 0.7865 | 0.3066 | 2.5652 | 0.0103 | 0.1856 | 1.3875 |
| I (Male % 61.86) | 0.0036 | 0.0132 | 0.2741 | 0.7840 | -0.0223 | 0.0295 |

**Moderator - Systolic blood pressure (Number of studies 8)**

|  | estimate | ci.lb | ci.ub |
| --- | --- | --- | --- |
| tau^2 | *1.2586* | *0.4446* | *10.2214* |
| tau | *1.1219* | *0.668* | *1.1971* |
| I^2 (%) | *97.4547* | *93.1156* | *99.6794* |
| H^2 | *39.2886* | *14.5256* | *311.9598* |

***R^2 = 0***

***QE = 34.7441; p-val = 4.830463e-06;***

***QM = 1.1909; p-val = 0.2751531***

|  | estimate | Se | z-val | p-val | ci.lb | ci.ub |
| --- | --- | --- | --- | --- | --- | --- |
| intercept | 0.7578 | 0.4233 | 1.7900 | 0.0735 | -0.072 | 1.5875 |
| I (SBP – 140.44) | 0.0364 | 0.0334 | 1.0913 | 0.2752 | -0.029 | 0.1018 |

**Moderator – Diastolic blood pressure (number of studies 7)**

|  | estimate | ci.lb | ci.ub |
| --- | --- | --- | --- |
| tau^2 | *2.0546* | *0.6624* | *18.4331* |
| tau | *1.4334* | *0.8139* | *4.2934* |
| I^2 (%) | *98.0164* | *94.0937* | *99.7749* |
| H^2 | *50.4131* | *16.9310* | *444.3127* |

**R^2= 0**

**QE = 33.9478; p-val = 2.438684e-06;**

**QM = 0.0694; p-val = 0.7922743**

|  | estimate | Se | z-val | p-val | ci.lb | ci.ub |
| --- | --- | --- | --- | --- | --- | --- |
| intercept | *0.8983* | *0.5822* | *1.5430* | *0.1228* | *-0.2427* | *2.0393* |
| I (DBP – 84.81) | *0.0205* | *0.0778* | *0.2634* | *0.7923* | *-0.1320* | *0.1729* |

**Moderator – HbA1c (Number of studies 10)**

|  | estimate | ci.lb | ci.ub |
| --- | --- | --- | --- |
| tau^2 | *0.7566* | *0.3002* | *4.9428* |
| tau | *0.8698* | *0.5479* | *2.2232* |
| I^2 (%) | *93.4733* | *85.0365* | *98.9425* |
| H^2 | *15.3217* | *6.6829* | *94.5592* |

**R^2 = 0;**

**QE = 37.4922; p-val = 9.341225e-06;**

**QM = 2.059; p-val = 0.1513115**

|  | estimate | Se | z-val | p-val | ci.lb | ci.ub |
| --- | --- | --- | --- | --- | --- | --- |
| intercept | *0.7275* | *0.2967* | *2.4523* | *0.0142* | *0.1461* | *1.3090* |
| I (HbA1c – 8.32) | *0.2732* | *0.1904* | *1.4349* | *0.1513* | *-0.1000* | *0.6463* |

**Moderator – Duration of diabetes (Number of studies 11)**

|  | estimate | ci.lb | ci.ub |
| --- | --- | --- | --- |
| tau^2 | *0.2307* | *0.1042* | *2.1258* |
| tau | *0.4803* | *0.3228* | *1.4580* |
| I^2 (%) | *87.4863* | *75.9518* | *98.4718* |
| H^2 | *7.9913* | *4.1583* | *65.4347* |

**R^2 = 50.4529**

**QE = 41.298; p-val 4.414324e-06**

**QM = 10.0856; p-val = 0.001494315**

|  | estimate | Se | z-val | p-val | ci.lb | ci.ub |
| --- | --- | --- | --- | --- | --- | --- |
| intercept | *0.4161* | *0.1689* | *2.4637* | *0.0138* | *0.0851* | *0.7472* |
| I (DurT2DM- 8.95) | *0.1937* | *0.0610* | *3.1758* | *0.0015* | *0.0741* | *0.3132* |

**Moderator – HDL cholesterol (Number of studies 3)**

|  | estimate | ci.lb | ci.ub |
| --- | --- | --- | --- |
| tau^2 | *9.9315* | *1.6624* | *100.0000* |
| tau | *3.1514* | *1.2893* | *10.0000* |
| I^2 (%) | *96.1972* | *80.8954* | *99.6089* |
| H^2 | *26.2967* | *5.2343* | *255.7107* |

**R^2 = 0**

**QE = 26.2967; p-val = 2.927947e-07**

**QM = 0.254; p-val = 0.6142553**

|  | estimate | Se | z-val | p-val | ci.lb | ci.ub |
| --- | --- | --- | --- | --- | --- | --- |
| intercept | *0.5221* | *2.9266* | *0.1784* | *0.8584* | *-5.2140* | *6.2581* |
| I (HDL – 1.43) | *-9.7731* | *19.3907* | *-0.5040* | *0.6143* | *-47.7781* | *28.2320* |

**Moderator – BMI (Number of studies 10)**

|  | estimate | ci.lb | ci.ub |
| --- | --- | --- | --- |
| tau^2 | *0.7363* | *0.3551* | *6.8630* |
| tau | *0.8581* | *0.5959* | *2.6197* |
| I^2 (%) | *94.5055* | *89.2410* | *99.3801* |
| H^2 | *18.2002* | *9.2945* | *161.3141* |

**R^2 = NA**

**QE = 32.8593; p-val = 6.529003e-05**

**QM = 0.0076; p-val = 0.9304357**

|  | estimate | Se | z-val | p-val | ci.lb | ci.ub |
| --- | --- | --- | --- | --- | --- | --- |
| intercept | *0.7256* | *0.2918* | *2.4865* | *0.0129* | *0.1537* | *1.2975* |
| I (BMI 27.12) | *0.0114* | *0.1310* | *0.0873* | *0.9304* | *-0.2453* | *0.2681* |

**Moderator – Triglyceride (TG) (Number of studies 5)**

|  | estimate | ci.lb | ci.ub |
| --- | --- | --- | --- |
| tau^2 | *0.0118* | *0* | *1.2811* |
| tau | *0.1088* | *0* | *1.1319* |
| I^2 (%) | *22.2732* | *0* | *96.8735* |
| H^2 | *1.2866* | *1* | *31.9849* |

**R^2 = 0**

**QE = 4.0654; p-val = 0.2544869**

**QM = 1.2989; p-val = 0.254408**

|  | estimate | Se | z-val | p-val | ci.lb | ci.ub |
| --- | --- | --- | --- | --- | --- | --- |
| intercept | *0.4269* | *0.0949* | *4.4982* | *0.0000* | *0.2409* | *0.613* |
| I (TG - 1.98) | *-0.1889* | *0.1658* | *-1.1397* | *0.2544* | *-0.5139* | *0.136* |

**Moderator - Total cholesterol (TC) (Number of studies 7)**

|  | estimate | ci.lb | ci.ub |
| --- | --- | --- | --- |
| tau^2 | *1.7422* | *0.5573* | *15.7757* |
| tau | *1.3199* | *0.7465* | *3.9719* |
| I^2 (%) | *97.9967* | *93.9926* | *99.7747* |
| H^2 | *49.9169* | *16.6461* | *443.9377* |

**R^2 = 0**

**QE 331513; p-val = 3.511879e-06**

**QM = 0.8426; p-val = 0.3586602**

|  | estimate | Se | z-val | p-val | ci.lb | ci.ub |
| --- | --- | --- | --- | --- | --- | --- |
| intercept | *0.9060* | *0.5156* | *1.7571* | *0.0789* | *-0.1046* | *1.9166* |
| I (TC – 5.56) | *1.1961* | *1.3031* | *0.9179* | *0.3857* | *-1.3579* | *3.7501* |

**MIXED EFFECT META-REGRESSION MODEL WITH DURATION OF T2DM**

Mean duration of T2DM across all studies: 10.14

Mixed-Effects Model (k = 11; tau^2 estimator: REML)

logLik deviance AIC BIC AICc

-9.6032 19.2064 25.2064 25.7981 30.0064

tau^2 (estimated amount of residual heterogeneity): 0.2307 (SE = 0.1389)

tau (square root of estimated tau^2 value): 0.4803

I^2 (residual heterogeneity / unaccounted variability): 87.49%

H^2 (unaccounted variability / sampling variability): 7.99

R^2 (amount of heterogeneity accounted for): 50.45%

Test for Residual Heterogeneity:

QE (df = 9) = 41.2980, p-val < .0001

Test of Moderators (coefficient(s) 2):

QM (df = 1) = 10.0856, p-val = 0.0015

Model Results:

estimate se zval pval ci.lb ci.ub

intrcpt 0.6457 0.1725 3.7423 0.0002 0.3075 0.9839 ***

mods 0.1937 0.0610 3.1758 0.0015 0.0741 0.3132 **

---

Signif. codes: 0 '***' 0.001 '**' 0.01 '*' 0.05 '.' 0.1 ' ' 1

**Supplementary table 2 – Relationship of duration of T2DM and the odds ratio of albuminuria. Statistical significance is indicated by italic font and asterisk (*)**

| Duration of T2DM | Odds ratio (OR) | 95% CI (lower limit) | 95% CI (upper limit) | p-value |
| --- | --- | --- | --- | --- |
| 4.00 | 0.58 | 0.28 | 1.20 | 0.14 |
| 5.00 | 0.71 | 0.38 | 1.31 | 0.26 |
| 6.00 | 0.86 | 0.51 | 1.44 | 0.55 |
| 7.00 | 1.04 | 0.67 | 1.61 | 0.86 |
| 8.00 | 1.26 | 0.87 | 1.82 | 0.21 |
| *9.00* | ***1.53*** | ***1.10*** | ***2.13*** | ***0.01**** |
| *10.00* | ***1.86*** | ***1.33*** | ***2.60*** | ***<0.001**** |
| *11.00* | ***2.26*** | ***1.54*** | ***3.29*** | ***<0.001**** |
| *12.00* | ***2.74*** | ***1.74*** | ***4.30*** | ***<0.001**** |
| *13.00* | ***3.32*** | ***1.94*** | ***5.70*** | ***<0.001**** |
| *14.00* | ***4.03*** | ***2.13*** | ***7.64*** | ***<0.001**** |
| *15.00* | ***4.89*** | ***2.32*** | ***10.30*** | ***<0.001**** |
| *16.00* | ***5.94*** | ***2.58*** | ***15.05*** | ***<0.001**** |


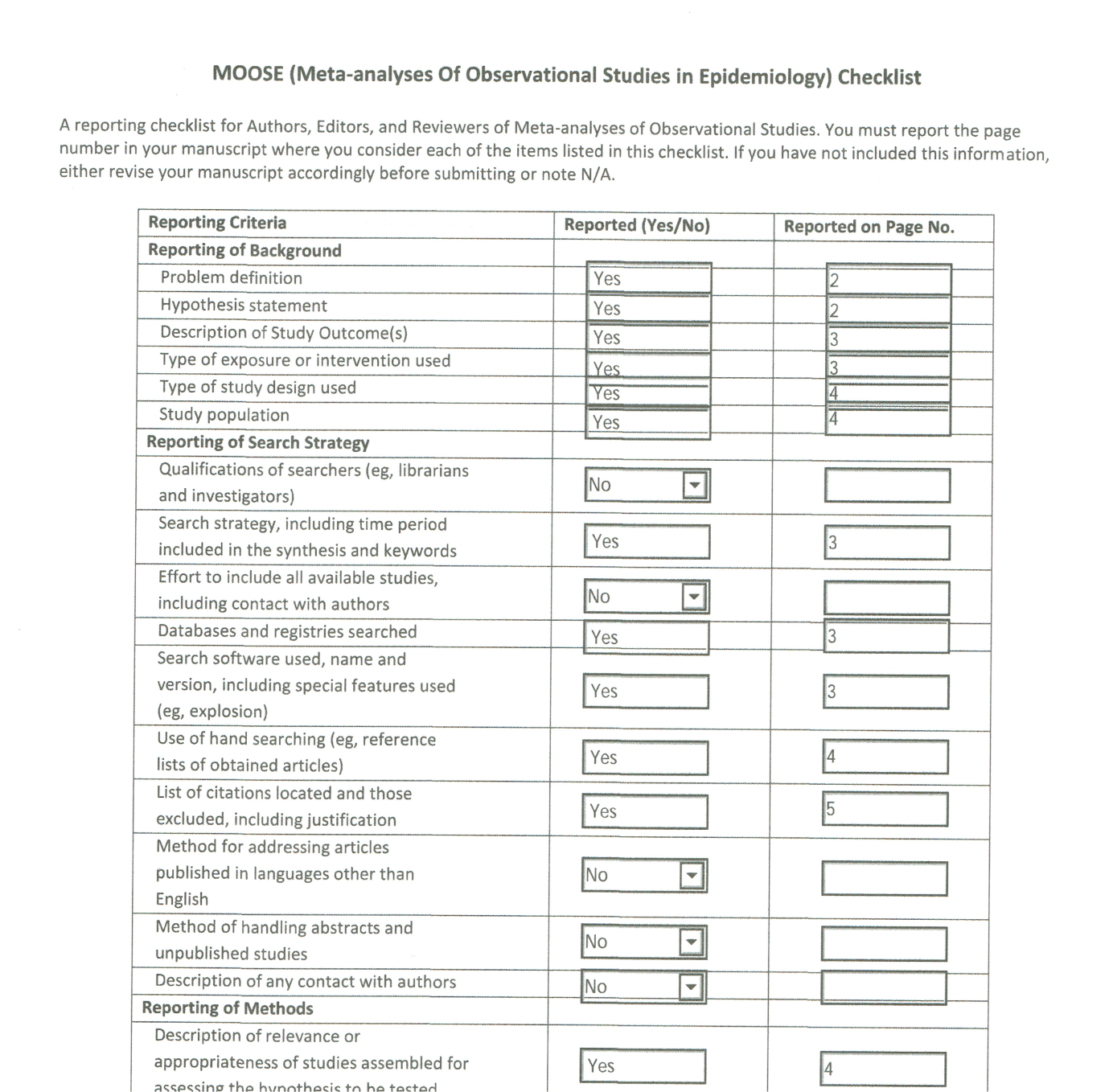

Supplement: Supplementary file 3 — Supplementary material 3 (DOCX 1190 KB) [file 592_2019_1293_MOESM3_ESM.docx]
